# Correction: Segura-Borrego et al. Influence of the Washing Process and the Time of Fruit Harvesting Throughout the Day on Quality and Chemosensory Profile of Organic Extra Virgin Olive Oils. *Foods* 2022, *11*, 3004

**DOI:** 10.3390/foods14213729

**Published:** 2025-10-30

**Authors:** M. Pilar Segura-Borrego, Rocío Ríos-Reina, Antonio J. Puentes-Campos, Juan G. Puentes-Campos, Brígida Jiménez-Herrera, Pedro Vallesquino-Laguna, Raquel M. Callejón

**Affiliations:** 1Área de Nutrición y Bromatología, Department de Nutrición y Bromatología, Toxicología y Medicina Legal, Facultad de Farmacia, Universidad de Sevilla, C/P. García González n°2, 41012 Seville, Spain; 2Servicio Promoción Rural, Delegación Territorial de Agricultura, Pesca, Agua y Desarrollo Rural en Granada, Avenida Joaquina Eguaras 2, 18013 Granada, Spain; 3University Institute of Research on Olive Groves and Olive Oils, GEOLIT Science and Technology Park, University of Jaen, 23620 Mengibar, Spain; 4Department of Technology, Postharvest, and Food Industries, IFAPA “Cabra” Centre, Institute for Research and Training in Agriculture and Fisheries, Ctra. Cabra-Doña Mencía, KM. 2.5, Cabra, 14940 Cordoba, Spain; 5Departamento de Bromatología y Tecnología de los Alimentos, ETSIAM, Universidad de Córdoba, Campus de Rabanales, 14071 Córdoba, Spain

## Figure/Table Legend

In the original publication [1], there was a mistake in the legend and note for Table 1. Sampling dates are not correct, and the note at the end of the table is unnecessary (must be removed). The correct legend appears below.
**Table 1.** Agronomic and physicochemical parameters of Picual Organic Extra Virgin Olive Oil (OEVOO) samples depending on the sampling day and the time of collection (morning or afternoon) and the washing process.

In addition, there was a mistake in the note added at the end of Table 2. The correct note appears below.
Note: Odor attributes assessed in a 1–10 scale. NW: Non-washed. W: Washed. M: Morning. A: Afternoon.

Likewise, there was a mistake at the end of the legend for Figure 2. The correct legend appears below.
**Figure 2.** Comparison of the sensory profiles (odor attributes) between the Organic Extra Virgin Olive Oil (OEVOOs) obtained from washed (W) and non-washed fruits (NW) (**a**) and from fruits harvested during morning (M) or afternoon (A) (**b**).

## Authors and Affiliations

This part of the paper requires several changes, as new authors and affiliations have been added or modified. For clarity, the updated list of authors and their affiliations, which must appear in the paper heading, is provided below:
**M. Pilar Segura-Borrego ^1^, Rocío Ríos-Reina ^1,^*, Antonio J. Puentes-Campos ^2^, Juan G. Puentes-Campos ^3^, Brígida Jiménez-Herrera ^4^, Pedro Vallesquino-Laguna ^5^ and Raquel M. Callejón ^1^**
^1^ Área de Nutrición y Bromatología, Department de Nutrición y Bromatología, Toxicología y Medicina Legal, Facultad de Farmacia, Universidad de Sevilla, C/P. García González n°2, 41012 Seville, Spain; msegura2@us.es (M.P.S.-B.); rcallejon@us.es (R.M.C.)^2^ Servicio Promoción Rural, Delegación Territorial de Agricultura, Pesca, Agua y Desarrollo Rural en Granada, Avenida Joaquina Eguaras 2, 18013 Granada, Spain; apuentescampos@gmail.com^3^ University Institute of Research on Olive Groves and Olive Oils, GEOLIT Science and Technology Park, University of Jaen, 23620 Mengibar, Spain; jpuentes@ujaen.es^4^ Department of Technology, Postharvest, and Food Industries, IFAPA “Cabra” Centre, Institute for Research and Training in Agriculture and Fisheries, Ctra. Cabra-Doña Mencía, KM. 2.5, Cabra, 14940 Cordoba, Spain; brigida.jimenez@juntadeandalucia.es^5^ Departamento de Bromatología y Tecnología de los Alimentos, ETSIAM, Universidad de Córdoba, Campus de Rabanales, 14071 Córdoba, Spain; bt1valap@uco.es*Correspondence: rrios5@us.es

## Author Contributions

As new authors have been added to the paper, the Author Contributions Section must be updated, as shown below.

**Author Contributions:** Conceptualization, B.J.-H., R.M.C. and P.V.-L.; Methodology, M.P.S.-B., R.R.-R. and P.V.-L.; Formal Analysis, M.P.S.-B., R.R.-R., A.J.P.-C., J.G.P.-C. and P.V.-L.; Investigation, M.P.S.-B., B.J.-H., R.M.C. and A.J.P.-C.; Resources, B.J.-H., R.M.C. and P.V.-L.; Data Curation, M.P.S.-B., R.R.-R., A.J.P.-C. and P.V.-L.; Writing—Original Draft Preparation, M.P.S.-B., R.R.-R., J.G.P.-C. and P.V.-L.; Writing—Review and Editing, R.M.C. and P.V.-L.; Supervision, B.J.-H., R.M.C. and P.V.-L. All authors have read and agreed to the published version of the manuscript.

## Acknowledgments Section

This part of the paper must be updated, to include the information shown below, as new companies and persons deserve to be recognized.

**Acknowledgments:** In the same way, they would also like to thank the company Alcanova S.L. for providing the raw material analyzed, and the Tasting Panel of the ‘Priego de Córdoba’ Denomination of Origin for their work on the sensory analysis of the samples studied. Finally, the authors would like to mention invaluable help from María Vergillos-Moreno and Rosario Sánchez-Marín (IFAPA “Cabra” Centre) for their collaboration in prior work related to this study.

## Abstract Correction

This section requires minor corrections due to the changes introduced in the paper. The revised paragraph is provided below: 

**Abstract:** In recent years, there has been a growing demand for organic extra virgin olive oils (OEVOOs) as quality products with greater added value. The aim of the present work was to determine whether the washing process and time of harvesting (morning or afternoon) plays an important role in the quality of Picual OEVOOs by studying quality parameters (degree of acidity, peroxide value, K_232_, K_270_, oxidative stability), and volatile and sensory profiles. With this in mind, olive fruits were harvested from an early stage of maturity (over a period of 32 days, during morning and afternoon hours), and then were analyzed considering two main blocks (washed and non-washed), each of which had two sample types (morning and afternoon). Among the analyses performed, it is noteworthy that the volatile profile was obtained by headspace solid-phase microextraction (HS-SPME) coupled to gas chromatography–mass spectrometry (GC-MS). Regarding the physicochemical quality parameters, as well as the sensory and volatile profiles, it is remarkable that there were no differences between the oils produced under the two treatments applied (washed/non-washed). However, the time of harvesting (morning or afternoon) did influence the volatile and sensory profile, with higher values in the oils obtained from fruits harvested in the morning, being statistically significant for the families of aldehydes, hydrocarbures, and lactones. Besides, the olives harvested during the mornings gave rise to oils with slightly higher values in the green and apple fruit attributes.

## Missing Citation and Text Correction

In the original publication [1], this reference was not cited (set now as [29] onwards):

Vallesquino-Laguna, P.; Puentes-Campos, A.J.; Puentes-Campos, J.G.; Jiménez-Herrera, B. Fruit washing influence on extra virgin olive oil quality: A sensory perspective. In *Actas del XIX Simposio Científico–Técnico del Aceite de Oliva—Expoliva*; Fundación del Olivar: Jaén, España, 2019; Volume IND–7, pp. 1–7. Available online: http://hdl.handle.net/10396/26332 (accessed on 18 October 2025).

In addition, this other one, which is related to the former, must also be considered (set now as [28] onwards):

Vallesquino-Laguna, P.; Puentes-Campos, A.J.; Puentes-Campos, J.G.; Vergillos-Moreno, M.; Sánchez-Marín, R.; Jiménez-Herrera, B. Influencia del lavado de frutos sobre determinados parámetros de calidad de un aceite de oliva virgen extra ecológico. In *Actas del XVII Simposio Científico Técnico—Expoliva*; Fundación del Olivar: Jaén, España, 2015; Volume IND–11, pp. 1–8. Available online: http://hdl.handle.net/10396/26334 (accessed on 18 October 2025).

These changes affect all references from [28] onwards in the original paper [1], which must now be set as follows: Original references [28–31] must be eliminated.

New references [28,29] should be the ones previously indicated.

New Reference [30] should be as follows: Council Regulation (EC) No 1804/1999 of 19 July 1999 Supplementing Regulation (EEC) No 2092/91 on Organic Production of Agricultural Products and Indications Referring Thereto on Agricultural Products and Foodstuffs to Include Livestock Production. Available online: https://eur-lex.europa.eu/eli/reg/1999/1804/1999-08-24 (accessed on 18 October 2025).

New Reference [31] should be as follows: Commission Regulation (EC) No 889/2008 of 5 September 2008 Laying Down Detailed Rules for the Implementation of Council Regulation (EC) No 834/2007 on Organic Production and Labelling of Organic Products with Regard to Organic Production, Labelling and Control. Available online: https://eur-lex.europa.eu/eli/reg/2008/889/oj/eng (accessed on 18 October 2025).

New Reference [32] should be as follows: Ferreira, J. *Collaborating Olive Farms No. 5*; Ministry of Agriculture: Madrid, Spain, 1979.

From reference [33] onwards, the following rule will be applied in the revised manuscript: each new reference [n] will correspond to reference [n−1] in the original article. For example, the new reference [33] will correspond to the old reference [32] in the original article, and the same applies to the following ones. Considering this, new reference [33] in the revised paper should be: Commission Regulation (EC) No. 640/2008 of 4 July 2008 amending Regulation (EEC) No. 2568/91 on the characteristics of olive oil and olive-residue oil and on the relevant methods of analysis. Available online: https://eur-lex.europa.eu/legal-content/EN/TXT/HTML/?uri=CELEX:32008R0640&from=ES (accessed on 30 June 2022).

These changes subsequently require several corrections throughout the text. The necessary revisions are indicated below:
**(I)** Introduction Section, end paragraph, at the beginning of page 3 in the original paper [1]. The text should read as follows:

In accordance with the above, it could be interesting to undertake an analysis that would delve into the influence of the washing process in the production of organic olive oil, as well as to study if the moment of harvesting influences the quality of OEVOOs. For this reason, and given the real need of producers to know whether these factors could have an important influence on the quality of the final OEVOO, research was initiated on this matter by the authors [28,29], showing initially that the washing operation alone did not significantly influence diverse physicochemical and sensory parameters of fruits harvested directly from the tree. Continuing with that research, the aim of this work is to determine if the washing operation, together with the harvesting moment, could both play an important role in a characteristic set of physicochemical, sensory, and volatile parameters of OEVOO. Taking into account the variety of analytes considered here, the results of this study could serve as a fundamental reference in this field for decision-making related to the harvesting and initial operations applied to olive fruits.
**(II)** Section 2.2 (“Olive Fruit Samples”), page 3 in the original paper [1]. The text should read as follows:

Olive fruits were collected from Picual olive trees cultivated in a certified organic dry-land farm since 2003 (by the Council Regulation (EC) No. 1804/99 [30] and Commission Regulation (EC) No. 889/2008 [31]), under organic crop production, located in Alcaudete (Jaén, Spain), which has a continental Mediterranean climate with dry summers and mild winters. As was described in [28,29], the plantation framework followed a traditional pattern (about 12 m × 12 m), and the fruits were only tree-harvested by using a vibration machine. The olive fruits were harvested very early, over a period of 32 days between October and November 2013, with an average ripening index (RI) close to 2.1 according to the method proposed by Ferreira et al. [32]. Samples were randomly taken in the field, and two batches of fruits (of around 15 kg each) were collected on the sampling days selected for this study (1, 3, 8, 15, 25, 29, and 32). Olives were not taken in any case from the ground since the intention was to obtain high-quality oils. In addition, the collection was carried out in the morning (from 8:00 to 12:00 am) and in the afternoon (from 13:00 to 15:00 pm) with the aim of studying how the harvesting moment through the day might affect the fruits. The batches of olives collected were later homogenized in the laboratory and split into two smaller samples (of 7.50 kg each) in order to have enough material to perform further analysis. During this stage, the characterization of the fruit was carried out by measuring the average weight of one hundred fruits and the RI.
**(III)** Sections 2.3 and 2.4 (“Olive Washing” and “Olive Oil Extraction”), pages 3 and 4 in the original paper [1]. The text should read as follows:

### 2.3. Olive Washing

From the samples collected in the morning, which were available each day of sampling, one of them was washed and the other one was left intact. The same was performed for the samples collected in the afternoon. The laboratory-scale washing process was undertaken using two 50 L drums, each containing 20 L of drinking water and the olives from a specific sample [28,29]. To simulate industrial washing conditions, compressed air from a regulation tank maintained at 600 kPa was injected into each drum for 2 min, promoting agitation of the olives in the water. The washing water was renewed every five days to approximately reflect the fluid conditions commonly present in industrial washing systems.

### 2.4. Olive Oil Extraction

Olive oil extraction was performed by using the Abencor system (Abengoa S.A., Sevilla, Spain) as was described in [28,29]. The oil produced from each sample was stored at 4 °C until analysis.

According to the experimental design, in this study, a total of 28 samples were processed, which were analyzed in duplicate. For the determination of the sensory and volatiles profile, a selection of samples was made, considering 3 stages of fruit maturity: an initial stage (day 1), an intermediate stage (day 15) and a final stage (day 32). Within each stage, these 4 types of samples were analyzed: washed-morning (WM), washed-afternoon (WA), non-washed-morning (NWM), and non-washed afternoon, (NWA).
**(IV)** Sections 2.5.1 and 2.5.2 (“Fruit Characterization” and “Analytical Indices”), page 4 in the original paper [1]. The text should read as follows for the “Ripening index” and “Analytical Indices” paragraphs: 

#### 2.5.1. Fruit Characterization


-Ripening index (RI): The method followed to determine the RI was that proposed by Ferreira et al. [32], which consists of classifying the fruit into eight classes according to the color of the skin and pulp.


#### 2.5.2. Analytical Indices

The indices free acidity, UV spectrophotometric indices (K_232_, K_270_), and peroxide values were evaluated, in triplicate for each sample, according to the official methods defined in the Commission Regulation (EEC) 2568/91 [11] and subsequent amendments of the Commission Regulation (EEC) 2568/91 [12,28,33].
**(V)** Section 3.1 (“Influence of Washing and Time of Harvesting on Agronomic and Physicochemical Parameters”), page 5 in the original paper [1]. The first paragraph, just preceding Table 1, should read as follows: 

### 3.1. Influence of Washing and Time of Harvesting on Agronomic and Physicochemical Parameters

Agronomic parameters such as weight of fruit and RI were determined to characterize the fruits (raw material). Then, physicochemical parameters such as free acidity, peroxide value, and UV spectrophotometric indices (K_232_, K_270_) and oxidative stability were determined as quality parameters to study if the fruit washing operation or even if the time of harvesting might have an important influence on the quality of the obtained OEVOOs (see Table 1).
**(VI)** Section 3.1 (“Influence of Washing and Time of Harvesting on Agronomic and Physicochemical Parameters”), pages 6 and 7 in the original paper [1]. The five paragraphs immediately following Table 1, should read as follows:

In line with preliminary research initiated by the authors [28], Table 1 provides a concise overview of the values of the parameters aforementioned for a selection of representative days (1, 3, 8, 15, 25, 29, and 32), which collectively cover the sampling period.

The average weight of 100 fruits was approximately 304 g, with a standard deviation of around 32 g. No correlation or dependence was observed between weight and time over the evaluation period, suggesting that growth had ceased before harvest and that the olives experienced no significant moisture loss or gain during sampling.

According to that table, the RI showed a clear upward trend for morning and afternoon samples, with minimum and maximum values of the order of 0.49–0.71 and 4.04–4.08. Bearing in mind the recommendations of some authors [37,38], who place the optimal time to start harvesting when the RI is close to 3.50, it is understood in this case that the harvesting tasks were developed in very early stages of maturation. However, recent studies [39,40] showed that for olive groves of the Picual variety, very early harvesting tends to be associated with better quality and stability of the oils. Similarly, according to other researchers [37,38], the fat yield over dry matter in the Picual variety is typically around or above 40% when the RI exceeds 2. This supports the harvest calendar normally chosen by the owners of the farm that supplied the samples for this study, as was initially suggested by the authors in [28].

The degree of acidity (%) of the non-washed (NW) samples was slightly higher than that of the washed ones (W), and the difference was statistically significant (*p*-value < 0.05). However, this difference could be considered negligible, as all samples were well below the maximum legal limit of 0.80% established for extra virgin oils. This suggests that the difference between both treatments (washing vs. non-washing) is not relevant. In addition, no significant differences were observed between the samples from fruits collected in the morning and those collected in the afternoon, regardless of the treatment.

The peroxide index, expressed as active meq O_2_/kg oil, initially showed a slight upward trend that stabilized at the middle and final stages of the sampling period. This evolution differs from that reported by Jiménez et al. [19] and Salvador et al. [41], who observed an inverse proportional relationship between the RI and the peroxide index, which they attributed to the increase in lipoxygenase activity. However, our results are in concordance with other authors [42]. On the other hand, it is noteworthy that the washing process and harvesting time did not affect this analyte. No significant differences were observed between the washed and non-washed samples, regardless of harvesting time (p-value > 0.05 according to the data in Table 1 and the preliminary results from [28]). In any case, the values of all the analyzed samples were quite far from the maximum legal limit of 20 meq active O_2_/kg oil, a fact that reflects that the oxidation state of the analyzed oils was very low.

The absorbance values in the ultraviolet region of the electromagnetic spectrum (K_232_ and K_270_) also did not show significant differences between washed/non-washed or morning/afternoon samples (see Table 1); practically all of them were below the legal limits for OEVOO (2.5 for K_232_ and 0.22 for K_270_), except for three cases linked to K_232_ (day 1, non-washed/afternoon, and days 3 and 25, washed/afternoon), which can be considered anomalous measurements as detailed in [28]. Additionally, the decreasing trend observed in the K_270_ values for both washed and non-washed samples is notable, in accordance with Jiménez et al. [19], which could be related to the chlorophyll content [19,39]. As the authors preliminarily stated in [28], this fact agrees with the values obtained for the RI of the samples, whose mean value was around 2.10. The presence of these pigments (chlorophylls) has been related to possible unfavorable evolutions in oxidative activity in oils [42,43]. However, given the oxidative stability time shown in the Rancimat analysis, 6–10 days (144–240 h) in almost all the samples (Table 1), it seems that, in our case, the presence of chlorophylls did not have a relevant effect, probably due to the conditions of conservation of the samples (refrigeration at 4 °C and preserved from light).
**(VII)** Section 3.1 (“Influence of Washing and Time of Harvesting on Agronomic and Physicochemical Parameters”), page 7 in the original paper [1]. Paragraph 3 (related to the “Rancimat results”) and the first sentence of paragraph 4 (related to the “washing and non-washing processes”) should read as follows:

The Rancimat results are noteworthy, since, although the fruits were collected in a state of early maturity, the organic oils of the Picual variety treated in this study, in addition to being of high quality, presented high values of oxidative stability, which is consistent with that reported by the authors in [28].

Considering the results presented in other works [40,44], the decreasing trend of oxidative stability time could be partially explained by the increase in the RI of the collected fruits. Regarding the washing process, unlike what happened with the degree of acidity, in the Rancimat tests, the non-washed samples presented a better performance than those that were washed, although these differences were not statistically significant (Table 1). Moreover, there is no marked difference in the values of this analyte between the morning and afternoon samples.

Hence, taking all the above into consideration, no relevant differences were observed, overall, in the physicochemical parameters just evaluated from samples treated by washing and non-washing processes. These results agree with those observed by Beltrán et al. [45], where no differences were found in the quality parameters between the washed and non-washed fruits stored in perforated boxes. However, Vichi et al. [46] observed lower values of the peroxide index and K_270_ in the oils obtained from washed and stored olives, possibly due to the anaerobic conditions that take place during storage. In our case, the olives were processed immediately after the washing process, so they did not have to be stored. Regarding the time of harvesting (morning or afternoon), no significant differences were observed in either any of the previously mentioned parameters, resulting, in all cases, in high quality oils (see also Figure 1a,b).
**(VIII)** Section 3.1 (“Influence of Washing and Time of Harvesting on Agronomic and Physicochemical Parameters”), paragraph 5 on page 7 (at the end of Section 3.1 in the original paper [1]). The first sentence of this part of the manuscript should read as follows:

In line with the aforementioned, and with the aim of complementing and analyzing previous work from a different perspective, a principal component analysis (PCA) was performed on all the parameters addressed thus far. Figure 1a,b (just shown) displays the scores and loadings plots, respectively, obtained by the first and third principal components (PCs), which explain 51.61% of the variance.
**(IX)** Section 3.2.1. (“Sensory Profile”), page 8 in the original paper [1]. The first 13 lines of the opening paragraph of this section, up to the text “(RI ranged between 0.49–4.08)”, should be rewritten as follows: 

Consistent with Section 2.4, Table 2 provides a compact summary of the sensory attributes of the OEVOOs previously analyzed by the authors in [29]. In this case, these parameters were evaluated at three stages of fruit maturity (initial stage—day 1; intermediate stage—day 15; and final stage—day 32) for both washed and non-washed samples collected in the morning and afternoon. It can be noted from the table that green fruity (average value AV = 5.30) was the most prominent sensory attribute under all conditions (washing/non-washing and morning/afternoon harvesting times). Other descriptors such as bitterness (AV = 4.00), pungency (AV = 3.50), and green leaf (AV = 3.30) were also notable, though to a lesser extent. The remaining attributes (fresh-cut grass, sweet, almond, apple, and tomato) were present in the OEVOOs tasted, but with lower intensity, as can be verified in Table 2.

These results are in agreement with the literature, since oils of the *Picual variety* are described as oils with “a great personality”, full-bodied, and with a high green fruity score for the olives—appreciating the taste of the olive leaf, slight itching and bitterness, that intensifies when the fruit is very green [24]. As was previously stated in [29], it is also remarkable that no defects were detected in the analyzed samples, resulting in an extra virgin classification in all cases. According to various authors [19,39,41,47,48], this outcome is common in fruits with low ripening indices, as is the case of the present study (RI ranged from 0.49 to 4.08), which would support the sensory evaluation results obtained.
**(X)** Section 3.2.1. (“Sensory Profile”), page 8 in the original paper [1]. The second paragraph of this section should be rewritten as follows:

For comparative purposes, the average attributes of the different types of samples considered in this study (WM, NWM, WA, NWA) are presented in a spider graph (Figure 2). Similarly to the results reported in [29], the sensory profiles of washed and non-washed samples were nearly identical, showing no significant differences in any of the sensory attributes analyzed with respect to the treatment applied to the fruits. However, regarding the harvesting time, some differences have been observed in the sensory profile of OEVOOs from fruits collected in the morning and in the afternoon. It is noteworthy that oils obtained in the mornings presented slightly higher values for the green fruity and apple attributes (see those marked with (*) in Figure 2b). These are positive attributes highly appreciated by consumers and, therefore, related to quality.
**(XI)** Conclusions Section, at the end of the third paragraph of this section (on page 13 in the original paper [1]). A minor change is required in the last sentence of this paragraph:

Regarding the sensory profiles, the olives harvested during the mornings gave rise to oils with slightly higher values in the green and apple fruit attributes.

## References

Throughout the text of this correction, several references have been mentioned. These citations are used and presented in the original paper [1], which is the subject of this revision and where they are properly documented along with other relevant studies. For clarity, and to avoid redundancy, only the citation from the revised manuscript [1] is displayed here. The authors state that the scientific conclusions are unaffected. This correction was approved by the Academic Editor. The original publication has also been updated.
