# Supplementary material for: Influence of the Washing Process and the Time of Fruit Harvesting Throughout the Day on Quality and Chemosensory Profile of Organic Extra Virgin Olive Oils
Source: Foods. 2022 Sep 27;11(19):3004. doi: 10.3390/foods11193004 (PMC9562684; doi:10.3390/foods11193004)
Supplement: Supplementary file 1 [file foods-11-03004-s001.zip › foods-1896925-supplementary.pdf]

Supplementary Material Segura-Borrego, et al.,

Table S1. Total Area of volatile compounds determined in organic Picual olive oil from washed olives by the HSSE-PDMS-GC-MS method.

				WASHED																
				Day 1					Day 15					Day 32					TT*	
**Compounds**	**LRI**	**ID**	**Odour Descr.**	**MORNING (WM1)**		**AFTERNOON (WA1)**		**T**	**MORNING (WM15)**		**AFTERNOON (WA15)**		**T**	**MORNING (WM32)**		**AFTERNOON (WA32)**		**T**		
				**Mean**	**±SD**	**Mean**	**±SD**		**Media**	**±SD**	**Media**	**±SD**		**Media**	**±SD**	**Media**	**±SD**		**M**	**A**
**Acetic acid esters**																				
Hexyl acetate	1247	A	Fruity	295318	9490	162483	87966	b	269551	29926	165826	228825	b	820213	22747	633566	84722	a		
(Z)-3-Hexenyl acetate	1291	A	Green	2234069	244982	1224258	574755	b	1645140	216040	777045	1313698	b	4123737	174669	3031396	701208	a		
**Acids**																				
Hexanoic acid	1841	A	Fatty	231558	117398	604222	346628		918927	154870	200350	389097		509981	397925	399836	707077			
Nonanoic acid	2168	B[1]	Waxy	657898	694488	690724	377810		676645	120820	407964	269276		344677	662294	737104	521137			
**Alcohols**																				
Methyl Alcohol	884	B[2]	Alcoholic	1169038	1055841	7230893	349024		1398755	167436	805153	6075672		631659	213016	1524508	526253			
Ethanol	912	A	Alcoholic	593238	167035	846285	93638	b	2743913	83174	639363	606854	b	3322023	684458	4145507	1830374	a		
1-Penten-3-ol	1138	A	Green	1212044	34675	555698	475776		876366	186475	424138	25426		469707	24510	400972	332032		*a*	*b*
													a							
2-Hexyn-1-ol	1200	B[2]	-	2372765	156086	1013047	1013624	a	1676786	500976	328715	788075	b	282231	14599	357329	960533	b		
(Z)-2-Penten-1-ol	1310	A	-	1915287	20855	885381	711537		1197800	183098	435050	200897		712397	34588	614156	556643		*a*	*b*
1-Hexanol	1345	A	Herbal, Green	443418	45296	225412	135629		464048	38173	1543780	357046		664828	21435	532903	752764			
(Z)-3-Hexen-1-ol	1374	A	Green	2062936	145145	1692921	186674		1699105	237851	7672215	902458		1519889	73836	1196197	4186705			
(E)-2-Hexen-1-ol	1396	A	Fruity	101330	31043	226368	106507		109753	13017	1348470	16299		75602	7462	65531	879632			
2-Furanmethanol	1654	A	Bready	2815666	603231	1449111	735841		1358478	1183802	1230065	546050		1694765	192640	1305560	187125			
1-Undecanol	1861	A	Waxy	1608656	303021	935589	819949		642526	243598	928441	6543		1523691	193320	728269	105474			
1-Dodecanol	1964	A	Waxy	2025033	990583	1600993	181878		1326333	866350	1490232	381742		953913	785634	1828283	276926			
Phenol	2003	B[2]	Phenolic	366227	107725	255883	49079		193444	115785	193267	28278		205388	57711	233056	29055			
2-Phenoxyethanol	2152	B[2]	Floral	374629	159926	160840	247203		180098	20039	175381	10671		157185	85454	185455	46081			
1-Hexadecanol	2376	B[3]	Waxy	412892	67847	450201	136947		370906	38147	588281	435823		482251	192452	743951	57478			
**Aldehydes**																				
Hexanal	1037	A	Green	3372282	62518	2674095	454086		4947488	202071	1741337	1114623		2965551	480975	2566368	2507582		*a*	*b*

3-Methyl-2-butenal	1089		Green	495564	3916	348175	126267		393348	69491	0	171998		147026	10900	162375	283589			
(Z)-2-Hexenal	1091	B²	Green	1027580	285962	367141	623033		856412	324	0	441398		113069	141546	124317	534802		*a*	*b*
(E)-2-Hexenal	1193	B²	Green	17699433	1008983	8346466	6056111		23355622	647163	1308938	621470		10864006	801967	8633047	15990347		*a*	*b*
Nonanal	1372	B¹	Aldehycic	3524212	921461	3706600	1197623		2712983	38109	3201342	573328		2560991	32196	2991517	329149			
Decanal	1484	A	Aldehycic	1358374	713879	1160936	488685		820568	113950	775398	222548		669749	80789	1078262	72338			
Benzaldehyde	1504	B²	Fruity	820631	294862	463061	100874		559334	59578	304478	135921		396236	47225	504171	203861			
(E)-2-Nonenal	1522	A	Fatty	214806	33869	194554	13842		250348	5898	140844	41677		153838	16988	172686	85928			
5-Methylfurfural	1559	B⁴	Caramellic	166169	11192	101310	48582		93412	21172	75635	25676		104156	3769	90185	14492			
(E)-2-Decenal	1641	B⁵	Waxy	392955	77307	466740	106925		478968	30456	362124	96141		338410	52334	435636	108834			
2-Undecenal	1740	B²	Fruity	488662	168115	838816	339024		710192	61269	539272	295988		536789	120645	761410	181185			
**Hydrocarbures**																				
Hexadecane	1586	B²	-	429722	140259	338239	50655		389239	10005	262600	164194		269068	147863	404252	163482			
Nonadecane	1886	B²	-	211381	86438	274010	42725		261718	28070	205012	166661		203274	118770	199419	99485			
(Z)-1-Methoxyhex-3-ene	965	B²	Green	1224610	7272	551063	115435		1413941	240137	749901	398145		1710065	32494	1260785	485798			
3-Ethyl-1,5-octadiene	982	B²	-	1654513	146	942976	475054		1496310	151900	592661	228026		581359	113353	533067	695656		*a*	*b*
Pentadecane	1489	C	-	572629	148520	302359	104544		308661	68373	240435	110327		273599	33125	296873	64809			
Heptadecane	1700	B²	-	475797	295609	461060	6513		453969	4140	239834	285907		280164	232375	373595	267643			
Octadecane	1800	B²	-	347781	242852	385546	11347		430862	64	233626	266701		249244	198560	232613	238750			
**Esters**																				
Isopropyl Myristate	2023	B²	-	1201802	240743	1138782	400607		977683	149612	1230700	1048223		898534	354080	1966556	1866			
Methyl palmitate	2203	B²	Waxy	412185	215546	597447	371834		537873	177280	454701	1460433		353577	282736	673172	200183			
Isopropyl Palmitate	2230	B²	-	371486	216699	799716	352164		482864	320684	574118	1013616		383012	287051	1282093	79003		*b*	*a*
Methyl dihydrojasmonate	2280	B²	Floral	514117	348233	621948	104915		1560915	92849	381831	138650		296125	286663	462158	977077			
**Ketones**																				
4-Methyl-2-hexanone	842	C	-	6760720	2731197	10046807	3929562	a	4606456	549724	3948662	532109	b	3096752	805502	6546884	864349	ab		
2-Pentanone	940	B²	Fruity	1486259	278974	840093	300902		749163	254601	6465266	514377		875565	47562	733593	4018110			
1-Penten-3-one	976	B²	Spicy	2274106	52591	1090078	846696		2085248	300348	20596	825553		970197	95331	948853	1507630		*a*	*b*
2-Octanone	1262	A	Earthy	555307	43983	723890	155198	a	205850	32883	145318	70839	b	147034	19417	163142	52515	b		

1-Hydroxy-2-propanone	1283	A	Caramellic	6536297	1513555	2799448	1946475		2544120	3148689	2686679	3002409		3264391	484389	2159632	141394			
6-Methyl-5-hepten-2-one	1316	A	Green	674811	139713	467830	74448	a	306942	26521	359247	49725	b	221123	19711	358359	27126	b		
1-Hydroxy-2-butanone	1358	B[2]	Coffe	463585	189319	155550	146216		149737	281080	176018	231412		222990	8355	120495	14369			
Acetophenone	1637	A	Floral	195514	17245	310622	184953		204335	17622	113176	97403		117712	108579	324547	118752			
1,2-Cyclopentanedione	1771	B[2]	Floral	1429999	10875	709764	621887		743416	673583	848281	193979		1012135	4082	665223	72106		*a*	*b*
2-Hydroxy-3-methyl-2-cyclopenten-1-one	1834	B[2]	-	336573	124835	150328	106605		130506	142938	152782	208142		181493	1763	112598	14867			
**Lactones**																				
5-Ethyl-2(5H)-furanone	1581	B[2]	-	741866	483466	273502	517351		620805	303247	0	333785		305461	34543	260149	471465		*a*	*b*
γ-Butyrolactone	1620	A	Creamy	767850	164385	319821	231698		297185	384848	281480	263856		351041	50105	282029	36161			
2(5H)-Furanone	1747	A	Buttery	1132047	108662	551985	380484		492142	485209	481938	254176		620351	63685	506154	39062			
2-Hydroxy-γ-butyrolactone	2189	C	Creamy	550441	113124	279571	145643		262984	240311	395388	185185		411501	71871	220339	57684			
**(Sesqui)Terpenes**																				
Copaene	1468	C	Woody, Spicy	155003	30830	76472	40791		103519	25571	124322	23393		105396	13214	75110	8066			
α-Farnesene	1724	B[2]	Woody, Green	167019	42082	111362	32466	b	248249	39441	133926	53485	a b	328618	68894	246392	115289	a		
**Others**																				
Pyrrole	1492	B[2]	Nutty	179452	58749	105071	25727		83696	51208	75933	26548		86256	13215	94149	12100			
Acetamide	1790	B[2]	-	207485	110355	160756	3900		90070	123688	85855	91597		98729	33406	175329	19863			

LRI: Experimetal Linear Retention Index values estimated by linear regression. ID: reliability of identification: A. mass spectrum and LRI agreed with standards; B. mass spectrum agreed with mass spectral data base and LRI agreed with the literature data; C. mass spectrum agreed with mass spectral data base. Odour Descriptor: information obtained from The good scents company (http://www.thegoodscentscompany.com). Literature reference agreed with LRI data: [1]da Silva, Freitas, Cabrita & Garcia, (2012) [63]. [2]National Center for Biotechnology Information (2005) [64]. [3]Liang, Chen, Reeves & Han, (2013) [65]. [4]Fan & Qian (2006) [66]. [5]Sales et al., (2019) [67]. Mean: mean area values. SD: standar deviation. TTdays: Tukey's test ($p<0.05$) between the morning and afternoon harvest for each day. Different lower case letters indicate significant difference between days (where a>b). T*: Tukey's test ($p<0.05$) among between the morning and afternoon harvest, regardless of the day. Different lower case italic letters indicate significant difference between days (where a>b). M: Morning; A: Afternoon.

Table S2. Total area of volatile compounds determined in organic Picual olive oil from non-washed olives by the HSSE-PDMS-GC-MS method.

				NON-WASHED																
				Day 1					Day15					Day 32						
**Compounds**	**LRI**	**ID**	**Odour Descr.**	**MORNING (NWM1)**		**AFTERNOON (NWA1)**		**T**	**MORNING (NWM15)**		**AFTERNOON (NWA15)**		**T**	**MORNING (NWM32)**		**AFTERNOON (NWA32)**		**T**	***TT***	
				**Media**	**±SD**	**Media**	**±SD**		**Media**	**±SD**	**Media**	**±SD**		**Media**	**±SD**	**Media**	**±SD**		***M***	***A***
**Acetic acid esters**																				
Hexyl acetate	1247	A	Fruity	272711	251	139295	311259	b	441100	6816	246392	488867	b	761543	88081	543043	216237	a		
(Z)-3-Hexenyl acetate	1291	A	Green	2031009	74	1029263	1590334	b	2896687	55636	1619090	2168711	ab	3900895	330117	2633383	565995	a	*a*	*b*
**Acids**																				
Hexanoic acid	1841	A	Fatty	170427	25154	290475	620709		291720	399001	353563	214882		195896	35120	343105	136464			
Nonanoic acid	2168	B[1]	Waxy	764275	3452	264623	432164		150058	7166	746147	257770		349076	414748	579778	340588			
**Alcohols**																				
Methyl Alcohol	884	B[2]	Alcoholic	1350368	312	1577160	318082		1386536	1982444	1071761	616015		788414	185513	453986	377204			
Ethanol	912	A	Alcoholic	1310486	7	16871965	3358745		4735263	66388	4365702	149442		4677828	257574	3510761	2159747			
1-Penten-3-ol	1138	A	Green	1122675	215	238794	82817		696469	8631	471587	52594		429824	36940	411558	497535		*a*	*b*
2-Hexyn-1-ol	1200	B[2]	-	2468968	694	150513	14545		1939985	44167	1188785	21903		259013	49452	228287	1529911			
(Z)-2-Penten-1-ol	1310	A	-	1812679	4512	466796	287046		978226	17790	703961	93273		723863	62904	664813	822646		*a*	*b*
1-Hexanol	1345	A	Herbal, Green	372282	541	1753531	705928		806984	40570	339223	144898		676352	59887	485353	91179			
(Z)-3-Hexen-1-ol	1374	A	Green	1989706	212	7826732	4436267		2304470	64028	1126244	70501		1515249	116941	1097073	566249			

(E)-2-Hexen-1-ol	1396	A	Fruity	93289	2556	1030591	902947		87004	23982	60663	31234		64671	4812	74751	14886			
2-Furanmethanol	1654	A	Bready	2691135	153	1029416	874351		1786234	362985	1409545	527101		1391741	347052	1169436	1153107		*a*	*b*
1-Undecanol	1861	A	Waxy	1651083	25	935526	91464		748492	1219650	1361458	918770		906768	176324	613402	528710			
1-Dodecanol	1964	A	Waxy	2250822	315	898601	399154		1134304	784599	1758153	565966		1167883	1158964	1823112	818425			
Phenol	2003	B[2]	Phenolic	385886	254	159627	116830		221135	51355	222397	46739		206128	79791	235637	185585			
2-Phenoxyethanol	2152	B[2]	Floral	264810	541	166256	3762		160273	50869	214590	66284		107814	24260	171112	72215			
1-Hexadecanol	2376	B[3]	Waxy	519243	62	447104	64775		181217	209188	497195	410469		279673	490060	451008	211515			
**Aldehydes**																				
Hexanal	1037	A	Green	3193183	4113	1787056	1597414		3596590	165614	2370628	1068446		2317005	276551	2161385	827926		*a*	*b*
3-Methyl-2-butenal	1089		Green	480584	2551	0	228347		345674	2711	264177	166928		160425	12036	155495	181961		*a*	*b*
(Z)-2-Hexenal	1091	B[2]	Green	1060727	21563	0	76049		906405	4858	521488	11747		88872	579	83908	641451			
(E)-2-Hexenal	1193	B[2]	Green	18847598	1553	1829105	4781636		17909314	69266	13831812	4364939		9671635	949465	7668852	7338040		*a*	*b*
Nonanal	1372	B[1]	Aldehycic	4127582	213	2736017	7620		3390059	847203	2570819	488121		2742919	1275648	2829201	1694032		*a*	*b*
Decanal	1484	A	Aldehycic	1478251	2215	501755	32690		1257069	443049	774034	351913		724693	632456	775658	519607		*a*	*b*
Benzaldehyde	1504	B[2]	Fruity	864647	22036	307295	236372		587416	43463	313535	47824		364141	103544	353636	375254		*a*	*b*
(E)-2-Nonenal	1522	A	Fatty	194983	1256	133905	45097	ab	227358	82180	169105	33564	a	106432	47125	133210	49381	b		
5-Methylfurfural	1559	B[4]	Caramellic	172149	2156	76802	59658		125721	17152	88139	26463		89281	31462	75270	82741		*a*	*b*
(E)-2-Decenal	1641	B[5]	Waxy	398320	3165	347408	173847	ab	439605	131928	421133	66162	a	253698	112135	358833	74444	b		
2-Undecenal	1740	B[2]	Fruity	600575	36102	547058	277141		532460	230159	773939	153452		401084	184757	649378	36536			
**Hydrocarbures**																				
Hexadecane	1586	B[2]	-	458767	51525	281097	28766		299437	129481	389569	80510		259892	191992	355409	91548			
Nonadecane	1886	B[2]	-	358358	22563	212570	10427		137968	56822	391233	22608		213343	79066	259292	175654			
(Z)-1-Methoxyhex-3-ene	965	B[2]	Green	1152245	2515	508003	202755		1562680	30470	883479	831556		1641580	218152	1145106	21804		*a*	*b*
3-Ethyl-1,5-octadiene	982	B[2]	-	1784845	154641	856464	39132	a	1278233	1043	884274	5874	ab	609768	48665	535637	966994	b		
Pentadecane	1489	C	-	487654	2113	193708	136904		394685	80205	277934	87477		255399	112696	235706	244316		*a*	*b*
Heptadecane	1700	B[2]	-	609552	222665	351088	74902		248438	125039	516580	59402		322259	142764	542013	144808			
Octadecane	1800	B[2]	-	567775	64565	316435	6388		184971	115802	555541	40977		292138	93150	419717	212415			
**Esters**																				
Isopropyl Myristate	2023	B[2]	-	1440100	225623	1029976	580205		600473	572424	1909365	479530		720464	405119	1371174	452704			

Methyl palmitate	2203	B^2	Waxy	711179	65526	840676	164666		275070	227348	875731	64498		444324	579250	500238	574298			
Isopropyl Palmitate	2230	B^2	-	398262	55621	645217	100770		214920	335896	882711	212862		285796	312652	1335852	282806		*b*	*a*
Methyl dihydrojasmonate	2280	B^2	Floral	949087	5211	537622	81832		368853	200000	940045	87035		339019	2548	635865	603101			
**Ketones**																				
4-Methyl-2-hexanone	842	C	-	11359800	65413	6362457	23188		4501480	313712	5483010	30584		4136400	2268634	3851985	7123572			
2-Pentanone	940	B^2	Fruity	1552972	3161	2369907	3739915	a	867812	43719	617944	26640	b	979447	179775	633952	798786	b		
1-Penten-3-one	976	B^2	Spicy	2138943	215633	29982	1097289		1706216	117175	1274142	529338		967179	139281	862830	873586		*a*	*b*
2-Octanone	1262	A	Earthy	689932	25512	1553877	89433	a	175132	19037	274717	21240	b	182901	51221	158793	404459	b		
1-Hydroxy-2-propanone	1283	A	Caramellic	5984501	55264	1842868	926278		3179873	552776	2588823	1421832		2940142	454847	2117855	3115667		*a*	*b*
6-Methyl-5-hepten-2-one	1316	A	Green	610627	25113	322527	80410		938578	68782	329175	52155		213820	62486	250881	203374			
1-Hydroxy-2-butanone	1358	B^2	Coffe	398299	5215	136902	69037		203147	30984	149891	72663		178719	12670	147475	177505		*a*	*b*
Acetophenone	1637	A	Floral	399024	525	129569	14647		211238	17761	179424	24250		112522	256227	128010	342171			
1,2-Cyclopentanedione	1771	B^2	Floral	1215705	2563	705180	228282		998439	347002	778302	372502		773824	3974	588377	393986		*a*	*b*
2-Hydroxy-3-methyl-2-cyclopenten-1-one	1834	B^2	-	280923	253	93050	65680		184442	61490	137816	87671		146854	2860	130129	105350		*a*	*b*
**Lactones**																				
5-Ethyl-2(5H)-furanone	1581	B^2	-	259302	25411	85354	972	b	739424	47535	465452	104604	a	210531	14063	64415	6849	b		
γ-Butyrolactone	1620	A	Creamy	686135	2551	196986	164330		381036	57538	305841	152004		321875	79398	203765	353858		*a*	*b*
2(5H)-Furanone	1747	A	Buttery	1085334	551	368114	312721		698299	145335	546069	212517		539666	128745	404765	569415		*a*	*b*
2-Hydroxy-γ-butyrolactone	2189	C	Creamy	506245	23512	191791	554		268159	66204	276196	243276		268029	54752	190055	261771			
**(Sesqui)Terpenes**																				
Copaene	1468	C	Woody, Spicy	159086	2255	73432	18850		116479	13473	98103	28219		85791	12268	66888	59553		*a*	*b*
α-Farnesene	1724	B^2	Woody, Green	182041	6314	86902	109696	b	197397	94272	184804	162959	ab	297967	21145	222196	52988	a		

**Others**																	
Pyrrole	1492	B[2]	Nutty	186217	2511	63826	57650	114980	25558	82748	30314	86332	33459	85888	89635	*a*	*b*
Acetamide	1790	B[2]	-	235761	52654	73295	129835	120587	39207	112836	31344	105404	100254	115034	91938		

LRI: Experimetal Linear Retention Index values estimated by linear regression. ID: reliability of identification: A. mass spectrum and LRI agreed with standards; B. mass spectrum agreed with mass spectral data base and LRI agreed with the literature data; C. mass spectrum agreed with mass spectral data base. Odour Descriptor: information obtained from The good scents company (http://www.thegoodscentscompany.com). Literature reference agreed with LRI data: [1]da Silva, Freitas, Cabrita & Garcia, (2012) [63]. [2]National Center for Biotechnology Information (2005) [64]. [3]Liang, Chen, Reeves & Han, (2013) [65]. [4]Fan & Qian (2006) [66]. [5]Sales et al., (2019) [67]. Mean: mean area values. SD: standar deviation. TTdays: Tukey's test ($p<0.05$) between the morning and afternoon harvest for each day. Different lower case letters indicate significant difference between days (where a>b). T*: Tukey's test ($p<0.05$) among between the morning and afternoon harvest, regardless of the day. Different lower case italic letters indicate significant difference between days (where a>b). M: Morning; A: Afternoon.
